# A novel *GABRB3* variant in Dravet syndrome: Case report and literature review

**DOI:** 10.1002/mgg3.1461

**Published:** 2020-09-18

**Authors:** Piero Pavone, Xena Giada Pappalardo, Simona D. Marino, Laura Sciuto, Giovanni Corsello, Martino Ruggieri, Enrico Parano, Maria Piccione, Raffaele Falsaperla

**Affiliations:** ^1^ Unit of Pediatrics and Pediatric Emergency University Hospital “Policlinico‐Vittorio Emanuele” Catania Italy; ^2^ National Council of Research Institute for Biomedical Research and Innovation (IRIB) Unit of Catania Italy; ^3^ Department of Biomedical and Biotechnological Sciences (BIOMETEC) University of Catania Italy; ^4^ Unit of Neonatology University Hospital “Policlinico‐Vittorio Emanuele” Catania Italy; ^5^ Department of Sciences for Health Promotion and Mother and Child Care “G. D’Alessandro” University of Palermo Italy

## Abstract

**Background:**

Mutations in *GABRB3* have been identified in subjects with different types of epilepsy and epileptic syndromes, including West syndrome (WS), Dravet syndrome (DS), Lennox‐Gastaut syndrome (LGS), myoclonic‐atonic epilepsy (MAE), and others.

**Methods and results:**

We herewith report on a girl affected by DS, who has been followed from infancy to the current age of 18 years. Next‐generation sequencing (NGS)‐based genetic testing for multigene analysis of neurodevelopmental disorders identified two likely de novo pathogenic mutations, a missense variant in *GABRB3* gene (c.842 C>T; p.Thr281IIe) and a nonsense variant found in *BBS4* gene (c.883 C>T; p.Arg295Ter).

**Conclusion:**

A likely relationship between the novel *GABRB3* gene variant and the clinical manifestations presented by the girl is proposed. Previously, one case of DS and two of DS‐like linked with *GABRB3* mutations have been reported. To the best of our knowledge, this is the first report of DS associated with this novel variant. A literature review of clinical cases with various types of epileptic encephalopathies (EEs) related to *GABRB3* mutations is reported.

## Abstract 2

Our study explores a likely new gene‐phenotype relationship between a novel GABRB3 gene variant and the clinical manifestations of Dravet syndrome (DS). We, herewith, report on a long‐term follow‐up of a girl affected by DS, who harbors a novel likely pathogenic variant of GABRB3. A literature review of cases with various types of epileptic encephalopathy related to GABRB3 mutations is discussed.

## 1 INTRODUCTION

The gene *GABRB3* (OMIM:137192) encodes gamma‐aminobutyric acid type A receptor beta‐3, a family member of the beta‐3 subunit of the gamma‐aminobutyric acid type A (GABAA) receptor, which is deputed to mediate inhibitory signaling within the central nervous system (CNS). GABAA receptor regulates through its physiological ligand GABA, the ion pore opens, thus allowing chloride to control influx or efflux. Reduction in the function of GABAA receptors may give rise to epileptic disorders (Hirose, 2014; Shi et al., 2019). Variants in GABAA receptor subunit, such as *GABRB3* located in chromosome 15q12, *GABRA1* (OMIM: 137160) and *GABRG2* (OMIM: 137164) located in chromosome 5q34, and *GABRD* located in chromosome 1p36.33, have been associated with several neurologic disorders including abnormal sensory processing, intellectual disability (ID), autism spectrum disorder (ASD), and seizures such as childhood absence epilepsy (CAE), juvenile myoclonic epilepsy (JME), febrile seizures (FS), generalized epilepsy with febrile seizures plus (GEFS+), and Dravet syndrome (DS) (Le, Le, Le, Kieu Huynh, & Hang Do, 2017; Macdonald, Kang, & Gallagher, 2010; Papandreou et al., 2016).

*GABRB3* mutations as cause of severe epileptic encephalopathies (EEs) were reported by Allen et al. (Epi et al., 2013) in subjects affected by West syndrome (WS) and Lennox‐Gastaut syndrome (LGS). Since then, approximately 30 cases have been described in subjects with severe epileptic seizures and different types of EEs. *GABRB3* mutations have been identified only in a few subjects presenting DS (Papandreou et al., 2016). DS is a severe type of epileptic seizures and belongs to the group of EEs, a wide and heterogeneous group of disorders presenting with various seizure types, interictal epileptiform discharges, and onset prevalently in early life (Berg, Tarquinio, & Koh, 2017; Gursoy & Ercal, 2016; Scheffer et al., 2017).

Here, were report a girl affected by DS presenting two de novo heterozygous mutations, a missense variant on *GABRB3* gene (c.842 C>T; p.Thr 281Ile) and a nonsense variant (c.883 C>T;p.Arg295Ter) on *BBS4* gene (OMIM: 600374). Previously, one case of DS and two cases of DS‐like linked to *GABRB3* mutation were described. To our knowledge, the present case is the first of a likely phenotype‐gene correlation between this novel *GABRB3* variant and DS. The girl has been followed up from infancy to the present age of 18 years. A review of the cases of EEs including DS linked to *GABRB3* variants is reported.

### 1.1 Case report

The girl is the second born of healthy unrelated Italian parents. The older sister is healthy. Family history is irrelevant with the exception of the father who had suffered by several episodes of FS up to the age of 4 years. The mother, at the time of gestation, was 27 years old and the father was 28 years old. The mother denied having had infectious diseases during her gestation and to have used drugs, alcohol, or toxic substances. Fetal movements were felt normally. The girl was born at term by programed cesarean section with the birth weight 2.900 g, height 50 cm, and occipitofrontal circumference (OFC) 35 cm (all values within normal limits). The Apgar score was 8 at 1 and 10 at 5 minutes. Perinatal period and developmental milestones were reached normally. At the age of 5 months, she was able to maintain the sitting position without support, the eye contact was present as well as the archaic reflexes. At seventh month, the girl complained of two episodes of FS lasting a few minutes. She came first at our observation at the Pediatric Clinic University of Catania, Italy at eighth month as she presented new episodes of FS of complex type with the characteristic of long durations and hemilateral prevalence. General and neurologic examinations were normal. She was able to stand up with support, she started lalling, and muscle tonus and strength were normal as well as patellar reflexes. Hematologic analyses showed at the Hb electrophoresis the presence of Hb A + F+A2, with HbF of 16.5% and Hb A2 of 4.20% consistent with a diagnosis of thalassemia carrier. The electroencephalography (EEG) showed bilateral slow waves mainly evident in the occipital regions. Treatment with phenobarbital was started. Some months later, other episodes of FS alternated with focal seizures, and myoclonic seizures were recorded. Seizures were resistant to drug treatment with phenobarbital and in the same time, a mild but progressively rapid motor and cognitive impairment was noted. At the age of 2.5 years, persisting the seizures, she was admitted again to this Institution. The seizures were of long duration, prevalently myoclonic and focal types. One of these episodes resulted in convulsive status epilepticus for which the admission of the girl to the emergency ward with intubation and appropriate treatment was necessary. The EEG pattern showed a slow background activity with polyspikes and wave discharges. The magnetic resonance imaging (MRI) of brain was normal. At this age, the neurologic examination revealed hypotonia with active patellar reflexes, developmental delay, and speech difficulty. Routine laboratory analysis including blood count, electrolytes, plasma and urinary amino acid, thyroid testing, organic acid, plasma purines, and total cholesterol were normal. No anomalies were found at the electrocardiogram (ECG) and cardiac ultrasonography. Genital organs were normal. The spleen and liver were palpated at the normal limits. Electromyography (EMG), nerve conduction velocity test (NCV), and ophthalmologic examination were also normal. Valproic acid was associated with phenobarbital with poor results. At the age of 7 years, next‐generation sequencing (NGS)‐based genetic testing was carried out. From the age of 7 years, stiripentol was added to valproic acid and phenobarbital with a progressive reduction of the frequency of seizures. She attended the primary school with teacher's aide with poor performance. She showed poor social contact with unknown people, but she was in friendship with her family and friends. A progressive increase of weight was noted. At 15 years old, she began menstruating with irregular cycles. A mild dysmorphism of the spine with dorsal hyperkyphosis was noted. The neurologic examination showed normal cranial nerves, with tendon reflexes symmetrical and normally elicited. Sensitivity was preserved. Neuropsychiatric evaluation showed an IQ of 42 on the Wechsler Intelligence Scale for Children (WISC‐III). The language was poorly expressive with scan sentences and words poorly structured. In the last 2 years under drug therapy, she had no more seizures. At the present age of 18 years, weight is 80 kg (<90th percentile), height 155 cm (10th percentile), and OFC 55 cm (50th percentile). She shows abundant panniculus adipose, thick and coarse hair, and youthful acne. Repetitive words, moderate ID, and ataxic gait have been noted. She shows reluctance to speech with unknown people. No seizures have been recorded. A new set of laboratory exams has given normal results. The brain MRI is normal. The EEG in wakefulness shows slow wave background, spike and waves mainly in the occipital regions of both hemispheres and during the sleep, the presence of slow waves intermixed with spike and waves with the tendency to generalize. Spindles have not been recorded. Abdomen ultrasound displayed cystic formations in both the ovaries, 50 cm in diameter on the right and 47 cm on the left.

### 1.2 Genetic testing and data analysis

Genomic DNA was isolated from peripheral blood of the proband and parents for array‐based comparative genome hybridization (aCGH) and NGS to perform genome‐wide copy number analysis and high‐throughput mutation screening. aCGH was performed using high‐resolution CytoSure ISCA 8x60 k microarray from Oxford Gene Technology (OGT) according to the manufacturers’ recommendation (Agilent Technologies). Data analysis was done using CytoSure software (GRCh38 assembly) provided by OGT. For the CNV interpretation were used publicly available patient data on Database of Genomic Variants (DGV) (dgv.tcag.ca), DECIPHER web‐based resource (decipher.Sanger.ac.uk), CNV dataset from Clinical Genome Resource (ClinGen), and Morbidity Map Developmental Delay. aCGH revealed no copy number alterations both in the proband and parents. Massive parallel sequencing was done on platform Ion Torrent PGM, Program Torrent Suite (Life Technologies). NGS data analysis was carried out with the program Ion Reported (Life Technologies). Panel used was Custom Panel Ion Ampliseq (AD 101847) including 376 amplicons containing the exonic and intronic regions adjacent to the sites of splicing (coverage mean 99.64%). Uniformity of the running coverage was 96.59% with a coverage 20X for 99.72% and with a coverage 100X for 99.09% of the total tracts.

Reference sequences used were NM_000814.6 (*GABRB3*) and NM_033028.5 (*BBS4*). NGS analysis identified two likely pathogenetic mutations, a heterozygous missense variant of the *GABRB3* gene (NC_000015.9:g.26806317C>T) (p.Thr281IIe; c.842 C>T) at chromosome 15q.12, and a heterozygous nonsense variant of the *BBS4* gene (NC_000015.9:g.73023914C>T) (c.883 C>T; p.Arg295Ter) at chromosome 15q24.1. The mutations were not found in both her parents. Prediction of pathogenicity of variants was determined by in silico tools such as PolyPhred, Sift, Provean, MutationAssessor, and MutationTaster. The revealed variants were confirmed by Sanger sequencing of products of amplification of DNA (ABI PRISM 3130XL Genetic Analyzer) and further data analysis with the program SeqScape v2.7 (Applied Biosystem).

## 2 DISCUSSION

The girl presented with episodes of seizures with high temperature started in first year of life and subsequently episodes of focal and myoclonic seizure types. In one of these episodes, the girl developed an episode of status epilepticus. The clinical manifestations, the course of the disorder, and EEG pattern led us to the diagnosis of DS. The girl has been followed up for several years till the present age of 18 years. At the last visit, moderate ID, language impairment, and difficulty to relate with other people except with the family were noted, whereas in the last 2 years no new seizures were registered. NGS results displayed two likely pathogenetic variants, a missense mutation in the *GABRB3* gene (c.842 C>T; p.Thr281IIe) and a nonsense mutation in the *BBS4* gene (c.883 C>T; p.Arg295Ter). Both her parents do not show these mutations. Up to date, the c.883C>T variant of *GABRB3 *has not been previously annotated in any genome variation database (i.e., gnomAD and 1000 Genome Project). Being a very rare de novo variant, data on allelic frequency are not yet available. In silico predictive analysis has indicated a disease potential of the amino acid substitution affecting a highly conserved protein domain located in the second transmembrane loop (M2) lining the intracellular side of the ion channel pore. Mutations in this protein region may negatively impact on receptor function, and the impaired protein function could also play a role in reducing Gabrb3 protein expression and its cellular trafficking (Papandreou et al., 2016). Recently, another variant p.Thr281Ala found in the same codon has been confirmed as pathogenic (Sterbova et al., 2018).

In assessing the stop‐gain mutation detected on *BBS4* gene, which codes for the fourth member of the Bardet‐Biedl syndrome (BBS) gene family (BBS; OMIM 615982) (Ece Solmaz et al., 2015), the variant (rs775710800) is extremely rare located in a semi‐conservative position, with a gnomAD frequency (0.00001), and causes the premature termination of the full‐length protein translation (519 aa). According to ACMG 2015 recommendations (Richards et al., 2015), the mutation identified is likely pathogenic. Still, the presence of a single heterozygous variant is not significant enough to establish the pathological phenotype of the BBS characterized by autosomal recessive inheritance. It is difficult, however, to postulate the exact pathogenic mechanism of the variant without any in vitro or in vivo functional work, thereby it may be deemed as a secondary genetic result, not clinically relevant in a phenotype‐gene relationship associated with the proband.

*BBS4* mutations have been associated with BBS, a well‐known disorder presenting with complex manifestations of cognitive impairment, truncal obesity, postaxial polydactyly, genitourinary malformations in female, and renal abnormalities. Rod‐cone dystrophy is seen at the ophthalmologic examination (Bardet, 1995; Biedl, 1995; Forsythe & Beales, 1993). In the proband, the impressive signs of BBS were not reported including the absence of postaxial polydactyly, ocular dystrophy, genitourinary, and renal malformations. Instead, *GABRB3* gene is well known to be associated with complex neurologic disorders including Rett syndrome (RS), Angelman syndrome (AS), ASD, and various types of epileptic disorders, including the EEs (Epi et al., 2013; Hamdan et al., 2014; Le et al., 2017; Moller et al., 2017; Myers et al., 2017; Papandreou et al., 2016). Among the EE group, de novo heterozygous mutations in the *GABRB3* gene are implicated in early infantile epileptic encephalopathy‐43 (EIEE43; OMIM 617113), an autosomal dominant seizure disorder. Le et al. (2017) in a study involving six DS subjects with *SCN1A*‐negative identified one subject presenting a de novo heterozygous missense novel variant c.695G>A (p.Arg232Gln) in *GABRB3*. These authors reviewed 13 cases of EEs from the literature correlated to *GABRB3* mutation with a total of 12 variants. Among these subjects, five had WS and LGS, one had epilepsy with myoclonic‐atonic seizures (Doose syndrome), six nonspecific EEs, and one DS‐like phenotype (Epi et al., 2013; Le et al., 2017). From the literature, we have selected and updated the cases of *GABRB3* variants related to the diagnosis of EE. Twenty‐six cases, including the present case, were collected: six were affected by MAE, five by LGS, four by WS, three by EE, three by EIEE, one by focal epilepsy, two by DS‐Like, and two by DS including our case (see Table 1). Reports of *GABRB3* variants in subjects with DS are very rare as one was reported by Le et al. (2017), the second is the present case, and other two cases have been reported as DS‐like.

**TABLE 1:** *GABRB3* variants related to the diagnosis of EE.

Author	No. Cases	Gender (M/F)	EE and syndrome	ID	Subjects examined
Epi et al. (2013)	4	M (n = 2); F (n = 2)	NS (n = 1); CGS (n = 3)	Severe (n = 3)	264
Hamdan et al. (2014)	1	M	EE	Severe	41
Papandreou et al. (2016)	1	M	FS	Severe	48
Myers et al. (2017)	7	F (n = 2)	EE (n = 2); EOEE (n = 2); MAE (n = 1); LGS (n = 1); DS‐like (n = 1)	Severe	531
Moller et al., (2017)	11	M (n = 8); F (n = 3)	MAE (n = 5); WS (n = 3); EOEE (n = 1); LGS (n = 1); DS‐like (n = 1)	Severe	416
Le et al. (2017)	1	M	DS (n = 1)	Severe	6
Present case	1	F	DS	Severe	1

Abbreviations: DS, dravet syndrome; EE, epileptic encephalopathy; EOEE, Early onset epileptic encephalopaty; FS, focal seizures; LGS, lennox‐gastaut syndrome; MAE, myoclonic‐atonic epilepsy; WS, west syndrome.

The present study represents the second observation after the case presented by Le et al. (2017) of the association of *GABRB3* variant with typical DS. In the proband, the diagnostic criteria were well represented including the family history of FS in the father, previous normal development, FS in the first year of life, myoclonic jerks and focal seizures, EEG pattern with polyspike and waves, and ID, ataxic gait, and poor response to treatment in the course of the disorder. In the case reported by Le et al. (2017), a first male child born to unrelated parents, the seizures started at 10th month with fever followed since the second year of life by various types of seizures, such as generalized tonic‐clonic seizure very sensitive to fever, myoclonic seizures, and complex partial seizures. In the case reported by Moller et al. (2017), the seizures started at eighth month and at the age of 2.5 years, mild developmental delay was noted and the EEG displayed bilateral spike and waves. The cases described by Moller et al. (2017) and Myers et al., (2017) were diagnosed as DS‐like since some criteria typical of DS were lacking.

The identified *GABRB3* mutation could be strengthened by the important role of GABA‐related genes associated with similar phenotype, as recently described for *GABRA3* (Niturad et al., 2017) and *GABRA1* (Johannesen et al., 2016) genes. Moreover, impairment of GABAergic pathway signaling is also supported by the largest epilepsy WES study to date, which highlights a ubiquitous role for GABAergic inhibition in epilepsy etiology (Feng et al., 2019). The father had several episodes of FS up to the age of 4 years with no recurrence of epileptic seizures. Genetic analysis has not disclosed the presence of mutations in the father, but the chance that he harbors *GABRB3* mosaicism may not be excluded. In view of the foregoing, according to the 2015 ACMG guidelines (Richards et al., 2015), the proband harbors a de novo likely pathogenic *GABRB3* variant, which corroborates recent findings on the DS candidate gene.

## 3 ETHICAL COMPLIANCE

The study was conducted ethically in accordance with the World Medical Association Declaration of Helsinki and was approved by the ethic committee of the University of Catania, Italy (Ethical Committee Catania 1 Clinical Registration n. 95/2018/PO). Informed consent was obtained from parents of the proband.

## CONFLICT OF INTEREST

The authors declare no financial or otherwise relevant conflict of interest related to this manuscript.

## AUTHOR CONTRIBUTIONS

PP and RF worked with and helped gather patient data, and drafted and redrafted the present manuscript. XGP and MP helped analyze the genetic data and interpret the literature relevant to the genomic imbalance. SDM made substantial contributions to the conception or design of the work, or to the acquisition, analysis, or interpretation of data for the work. GC contributed to the clinical understanding of the case. LS, MR, and EP were called for their specialist consultancy regarding the clinical diagnosis and for reviewing the final version of the work. All authors read and approved the final manuscript.

## Data Availability

The data used to support the findings of this study may be released upon application to the corresponding author who can be contacted at ppavone@unict.it.
